# Overcoming the struggle of living with type 2 diabetes – diabetes specialist nurses’ and patients’ perspectives on digital interventions

**DOI:** 10.1186/s12913-023-09277-y

**Published:** 2023-03-30

**Authors:** Frida Jarl, Anna Davelid, Katarina Hedin, Andreas Stomby, Christina Petersson

**Affiliations:** 1 Rosenhälsans vårdcentral, Region Jönköping County, Jönköpingsvägen 19, Huskvarna, SE-551 85 Sweden; 2 Department of Health, Medicine and Caring Sciences, Linköping University, Linköping, Sweden; 3 Futurum, Region Jönköping County, Jönköping, Sweden; 4 Råslätts vårdcentral, Region Jönköping County, Jönköping, Sweden; 5 Center for Learning and Innovation, Region Jönköping County, Huskvarna, Sweden; 6 Jönköping Academy for Improvement of Health and Welfare, Jönköping University, Jönköping, Sweden

**Keywords:** Type 2 diabetes, primary health care, Self-management, Qualitative research, Digital health intervention, Diabetes self-management education and support

## Abstract

**Background:**

Diabetes self-management education and support (DSMES) is a cornerstone in the treatment of type 2 diabetes mellitus (T2DM). It is unclear whether delivering DSMES as a digital health intervention (DHI) might meet the needs experienced by patients with T2DM and diabetes specialist nurses (DSN) of the primary health care system in Sweden.

**Methods:**

Fourteen patients with T2DM and four DSN participated in three separate focus groups: two groups comprised patients and one group comprised DSN. The patients discussed the questions: “What needs did you experience after your T2DM diagnosis?” and “How might these needs be met with a DHI?” The DSN discussed the questions: “What needs do you experience when treating a patient with newly diagnosed T2DM?” and “How might these needs be met with a DHI?”. Furthermore, data were collected in the form of field notes from group discussions at a meeting including 18 DSNs working with T2DM in PHCCs. The discussions from focus groups were transcribed verbatim and analyzed together with the field notes from the meeting using inductive content analysis.

**Results:**

The analysis yielded the overall theme: “Overcoming the struggle of living with T2DM”, which was summarized in two categories: “learning and being prepared” and “giving and receiving support”. Important findings were that, for success, a DHI for DSMES must be integrated into routine care, provide structured, high-quality information, suggest tasks to stimulate behavioral changes, and provide feedback from the DSN to the patient.

**Conclusion:**

This study highlighted several important aspects, from the perspectives of both the patient with T2DM and the DSN, which should be taken into consideration for the successful development and use of a DHI for DSMES.

## Background

The global prevalence of type 2 diabetes mellitus (T2DM) has continued to increase, which has resulted in a considerable burden of morbidity and mortality [1]. Diabetes-related complications contribute to the adverse impact that T2DM has on quality of life and increasing healthcare costs [2]. Therefore, it is necessary to find management strategies that focus on improving quality of life, minimizing the risks of complications, and improving care [2, 3].

The goal of diabetes self-management education and support (DSMES) is to support a person’s ability to take responsibility for their own behavior and well-being. DSMES was shown to prevent or delay the complications of T2DM [4]. DSMES places the responsibility on the individual to cope effectively with symptoms, treatments, and lifestyle changes [5]. T2DM affects several aspects of life, and lifestyle changes are a cornerstone in the treatment process. Previous research has demonstrated that access to DSMES is important for people with T2DM, because it supports high-quality diabetes care [6]. DSMES is traditionally offered through in-person educational programs, but that approach is resource intensive [7].

Advances in mobile technology have provided an opportunity to deliver DSMES to patients with a digital health intervention (DHI) that may be convenient and potentially cost-effective [8, 9]. It has been suggested that the success of a DHI is linked to how well it relates to the patient’s routine clinical care [10]. Moreover, a DHI may have a greater impact on patients with T2DM, compared to care as usual, by promoting favorable lifestyle and eating habits [11]. Thus, a DHI that provides DSMES may provide better than usual care by improving important health factors. However, the key factors for achieving positive long-term effects remain unclear and further studies of the effects of the interventions on other parameters than glucose control are needed [12].

A fundamental aspect of improving usual care is to address the needs of both the patients and the healthcare providers. Therefore, it is important to gain more knowledge about the needs experienced by patients with T2DM and by diabetes specialist nurses (DSNs) [13].

Most previous research in this area has focused on either the patient or DSN perspective; thus, studies that integrate aspects from both sides are lacking [14, 15]. Notably, only 19 of 47 countries in Europe recognize diabetes nursing as a specialty and provide diabetes-specific education for nurses [16]. Furthermore, in Sweden, DSNs working in primary healthcare (PHC) play a prominent role in providing care for patients with T2D. Thus, it may be complicated to transfer knowledge on the use of DHIs from other healthcare systems to a Swedish setting [17].

The present study aimed to explore patient needs after being diagnosed with T2DM, the needs of DSNs, treating these patients, and to elucidate how these needs identified by patients and DSNs might be met by a DHI for DSMES.

## Methods

### Setting

This exploratory qualitative study was conducted in a medium-sized city in the southern part of Sweden, with 140,000 inhabitants, in November 2019. The patients were recruited from two primary healthcare centers (PHCCs), where the authors (AS, FJ, AD) worked as resident physicians. The PHCCs had a total of 30,000 listed patients of all ages and of both Swedish and foreign backgrounds. The average prevalence of type 2 diabetes in the primary health care in region Jönköping County is approximately 6%. The DSNs were recruited from four different PHCCs in the region. Furthermore, all DSNs that specialized in diabetes and worked in the local PHCCs were invited to attend a yearly networking meeting, where data was also collected. All patients and DSNs received written information before entering the study, and all provided written informed consent before data collection started.

### Patient focus groups

The Swedish National Diabetes Registry (NDR) was accessed to identify patients under 80 years old that had been diagnosed with T2DM within three years without insulin treatment in each PHCC. Approximately 80 patients met these criteria. The researchers contacted these patients by phone with the aim of recruiting a sample with maximum variation in age, sex, ethnicity (based on name), and HbA1c level. All these data were registered in NDR, and they were collected in a clinical research form after the participants provided written informed consent to participate. Patients were excluded when they did not speak Swedish or experienced other diseases that prohibited participation in a focus group. This information was gathered during the phone call. A total of 14 patients from the list were interested in participating, and all 14 entered the study after providing written information. One participant had been diagnosed with T2DM more than three years prior to participating in a focus group, but we decided to retain that patient in the study.

The 14 patients with T2DM attended one of two focus groups (n = 6 and n = 8). All interviews took place at the two PHCCs described above, and the patients were allowed to choose the focus group they wished to attend, based on their availability for a daytime or evening meeting.

### DSN focus groups

Four DSNs employed at four different PHCCs known by the researchers were contacted by e-mail and phone and agreed to participate in a third focus group. The four DSNs attended a separate focus group.

### Structure of the focus groups

Each focus group included two researchers; one acted as a moderator (FJ or AD) and one acted as an observer (FJ, AD, or AS). An interview guide including two open main questions and subsequent probing questions to help participants provide deeper answers was followed. In the focus group with DSN the first open question to discuss was: “What needs do you experience when treating a patient with recently diagnosed T2DM?”. In the two focus groups with patients the first question was: “What needs did you experience after your T2DM diagnosis?”. The same second question was used in all focus groups: “How might these needs be met with a DHI?”. Each focus group session lasted about 90 min. The observer took field notes, which were used to summarize the discussion. All uncertainties were clarified at the end of the session. The focus groups were arranged to avoid doctor-patient or professional-layman attitudes between the moderator and the patients or DSNs i.e. no doctor-patient relationship existed between the participants and the interviewer. The discussions from all three focus groups were audio-recorded and transcribed verbatim [18].

### The networking meeting with DSNs

Furthermore, data were collected at the yearly regional networking meeting for DSNs that worked with T2DM in the PHCC. Participants at the meeting were informed verbally that participation was voluntarily, and that they could choose not to participate. Eighteen DSNs were randomly grouped into five groups to discuss the question: “How could an internet-based tool for diabetes treatment improve your ability to help patients with T2DM?”. The groups took field ntes during the discussions, which lasted for about 20 min. At the end, each group presented their thoughts verbally to the other groups. The researchers (FJ, AS and AD) collected the written notes from each group, which were later used to summarize input from the discussions.

### Data analysis

An integrated analysis of all data from the focus groups and the networking meeting was performed in several steps with inductive qualitative content analysis [19]. First, the content from the focus groups was read several times by FJ and AD to familiarize with the data. Then, parts of the text answering to the aim of the study were collected and condensed into smaller units, which were shortened to codes. Then, codes were devised to illustrate the main topics, and each code reflected the corresponding focus group. The short written notes from the networking meeting were also encoded. Next, the codes were compared, sorted, and merged into similar and dissimilar subcategories. This process included codes from patients and DSNs from the focus groups and DSNs from the networking meeting. This process was supervised by an experienced researcher in qualitative methods (CP) that was not involved in the data collection. The subcategories were discussed between all the investigators to reach a consensus. Next, the subcategories were abstracted into two generic categories. Finally, the generic categories were abstracted into a main theme, which described the needs experienced by the participants. To achieve reliability, all subcategories, generic categories, and main categories were discussed among all researchers. Adjustments were made during these discussions, where the researchers turned back to the data to confirm that the interpretations were in accordance with the data.

## Results

A total of 14 patients agreed to participate and were included in the first two focus groups (Table 1). The four DSNs in the third focus group were female, their median age was 57 years (range 31–62), and they had been working as a DSN for a median of 15 years (range 4–19). All but one of the 18 DSNs that participated in the regional networking meeting were female, and they had been working with patients with diabetes for a median of 6.5 years (range 0–23).

**Table 1:** Characteristics of the 14 included patients

Median age, years (range)	60 (41–78)
Median diabetes duration, years (range)	2 (0–8)
Educational level, n (elementary school/college/university)	2/5/7
Smartphone or computer use, n (monthly/ weekly/daily)	0/0/14
Ethnicity, n (Swedish/foreign)	12/2
Diabetes treatment, n (Diet/oral drug/insulin)	1/13/0

### Overcoming the struggle of living with T2DM

The results of this study are presented and discussed thematically, in terms of two generic categories and four subcategories. A summary is given in Table 2. The overall theme, *“Overcoming the struggle of living with T2DM”*, was interpreted from the patient perspective as striving towards the goal of living a desirable life, despite T2DM. From the DSN perspective, the overall theme was interpreted as striving to support patients to facilitate living their lives, despite T2DM. The two generic categories were: *“Learning and being prepared”* and *“Giving and receiving support”*. Each generic category included two subcategories that were shared by the patients and the DSNs.

**Table 2:** Summary of main findings

Theme	Overcoming the struggle of living with T2D							
Generic categories	Learning and being prepared				Giving and receiving support			
Subcategories	Understanding		Control and responsibility		Feedback		Timing	
	*Patients*	*DSN*	*Patients*	*DSN*	*Patients*	*DSN*	*Patients*	*DSN*
Needs:	Need to know more about diabetes, in general, and healthy habits, in particular; need to understand and accept negative emotions linked to the diagnosis of T2DM	Need to provide clear and correct information about T2DM; need to understand the patient’s unique situation to provide person-centered care	Want to take control by measuring blood glucose and other parameters; transfers responsibility regarding life style to the DSN	Want the patient to take responsibility for their disease; self-care should be supported by healthcare providers	Essential to get feedback from the DSN regarding lifestyle and drug treatment; want individualized advice and regularly meetings with the DSN	Want to give personalized feedback more regularly than that given during physical visits; want to promote healthy behaviors instead of providing basic facts	Need time to process information about T2DM and to prepare before visits; want feedback immediately, when needed	Need time to learn and implement new digital strategies; want to give feedback more regularly over a longer time period
	**How these needs might be met with a DHI**							
	*Patients*	*DSN*	*Patients*	*DSN*	*Patients*	*DSN*	*Patients*	*DSN*
Solutions:	High quality on-line information available when the patient wants it; peer support can facilitate understanding; Brief information adapted to all patients with pictures and video clips	Give basic, high quality information before healthcare visits; give structured information on healthy habits, such as recipes; use checklists to increase understanding	Motivate the patient by providing tasks that focus on behavioral change; increase understanding to support responsibility	Transfer responsibility to the patient by increasing understanding and providing more support for healthy behaviors; make measurement data accessible to DSN	Provide a platform to receive regular feedback on behavioral changes from the DSN; need the DHI to be integrated into routine care	Provide a new platform to give regular feedback to the patient over time; provide more information on patient’s lifestyle to give person-centered feedback	DHI available whenever the patient wants to provide information and feedback at the right time; DHI can be used where and when the patient needs it	DHI should prepare the patient before physical visits, so DSN can spend time on supporting behavioral changes and more time on patients with greater needs

*“Overcoming the struggle of living with T2DM”* reflects several important aspects highlighted by patients and DSNs in the focus groups. One aspect relates to achieving and sustaining healthy habits over many years, even though most patients have few symptoms. The DSNs struggled to help patients understand how diabetes affects the body and how to prevent complications. They wanted to transfer more responsibility to the patient, partly because DSNs cannot take responsibility for a patient’s health and disease, and partly because DSNs lack time, due to heavy workloads. Another aspect of the theme relates to the need to overcome feelings of insecurity and anxiety, which required reassurance and control. This need was illustrated as allowing patients to take more control through, e.g., blood glucose testing. The last aspect of overcoming the struggle of living with T2DM included how to deal with the emotions evoked by being diagnosed with T2DM. The diagnosis puts a lot of pressure on most patients, and emotions such as shock, anger, and denial are representative of the struggle. Three citations illustrated the struggle from patient and DSN perspectives:

*”I was told about the diagnosis during a visit. I became angry and shocked; hard to come up with questions then” (Patient, focus group 1)*.

*“If my wife eats a nut, her throat swells instantly. If I eat a piece of candy. I don’t notice anything.” (Patient, focus group 2)*.

*”[I often struggle] to help the patients understand that it is up to them, and not me, to make them change” (DSN, focus group 3)*.

### Learning and being prepared

Learning and being prepared highlights the patient’s need to gain knowledge about the disease to facilitate living every day with diabetes. Furthermore, patients must use that knowledge to become prepared and to act according to their knowledge. From the DSN’s perspective, this category illustrated the need to learn about the patient, which facilitated providing person-centered care, and being prepared to support the patient’s specific needs.

*“At the first visit, it would be great [to get] some examples of things to ask. I did not even know what to ask” (patient, focus group 1)*.

*”I always start by asking them what they know about diabetes. Important to know who sits in front of you to know where to start“(DSN, focus group 3)*.

#### Learning and being prepared: understanding

The patients clearly expressed the need for knowledge about appropriate lifestyle choices. The discussions focused primarily on the need for more knowledge about how to achieve a healthy diet and how to choose suitable foods. They also asked for more information about physical activity. They wanted clear, balanced information about their disease and how it might affect the body, so they could be prepared without worrying about future unknown complications. Furthermore, patients expressed the need to understand how this knowledge could be applied to their everyday life; e.g., how to act during special occasions, like traveling or going to a party. Their goal was to be able to manage unpredictable situations.

*“I know that it [the diabetes] wears on the internal organs. You don’t really know how and what to think about” (Patient, focus group 2)*.

*“[I need] detailed, written dietary advice. What the doctor says quickly disappears.” (Patient, focus group 2)*.

The DSNs described the need for information about T2DM from reliable sources, to ensure they gave their patients the correct information. The DSNs wanted to focus on motivating the patients to act and use knowledge when they changed behaviors and encountered specific situations, rather than just repeating the same basic information.

According to the patients and the DSNs in this study, understanding could be met with a DHI for DSMES by using structured, reliable information about: diabetes in general, potential complications, and healthy foods. Information about healthy foods should contain facts about sugar and carbohydrates, recipes, and a quantification method for determining how much to eat. Understanding could be facilitated by using illustrative pictures and informative films, which should be available in several languages. Another way of achieving understanding would be to prepare the patient before each medical visit; e.g., providing information about the visit, suggestions about questions to ask the DSN, and reading material to learn medical facts about T2DM.

*”The need for knowledge appeared immediately after receiving the diagnosis, to get a thorough explanation about what happens in the body when you have diabetes“ (patient, focus group 1)*.

*“If they could read about dietary recommendations, I would not have to spend time providing this information during the visit. Then, I could focus more on patients with poor glucose control“ (DSN, focus group 3)*.

However, before the patient can achieve understanding, they need to overcome the emotional aspect of being diagnosed with diabetes. This need might be met by including a section on different aspects of living with a chronic disease, working toward acceptance by dealing with potentially blocking emotions, and also, by developing a persona for a deeper understanding about living with diabetes. Overcoming the emotional impact is particularly important at the time the patient receives a diagnosis, according to both patients and DSNs.

*“I became very surprised. I didn’t take it [the diabetes diagnosis] to heart. The doctor called after the weekend. I had managed to digest it a bit by which time” (Patient, focus group 2)*.

#### Learning and being prepared – control and responsibility

The patients in the focus groups shared a sense of insecurity, because diabetes seldom causes symptoms, in the short term. Most patients agreed that the solution to this insecurity was to measure blood sugar levels, blood pressure, and physical activity levels (with a pedometer) more often. Frequent measurements was a strategy for taking control and responsibility. Another aspect of being in control of the disease was knowing that someone was supervising the tasks and lifestyle changes made by the patient. Patients thought it was easier to adhere and maintain their new habits when a DSN or a physiotherapist was supervising them. It made them feel like they were doing good, when they were considered more adherent.

*“We need to come up with a way to analyze and measure, we cannot improve, if we do not measure” (patient focus group 1)*.

*“I had a blood pressure monitor at home and it was great fun writing it up. I have still saved that paper.” (Patient, focus group 2)*.

On the other hand, the DSNs wanted many of their patients to take on more responsibility for the disease. They felt that they had to remind their patients too often, and that patients lacked an understanding of the seriousness of diabetes. According to the DSNs, controlling blood glucose might be one way to encourage patients to take responsibility for their T2DM, but they were against controlling without a purpose.

*”We have a lot to gain, if we make them take care of their own disease. I think we are taking over that responsibility a bit too much”(DSN, focus group 3)*.

Another aspect of control and responsibility, from the DSN’s point of view, was that they felt responsible about the information given to the patients. They wanted to ensure it was correct and credible.

To create a DHI that met the needs of control and responsibility, both patients and DSNs suggested that it must document recurrent tasks. For example, the DHI must accommodate forms for recording physical activity, food diaries, and frequent reports on blood glucose levels. DSNs suggested that data reported by the patient could provide a deeper understanding about the patient’s disease and needs.

*“[I want a] exercise module. To fill in their activity – get feedback in the meantime” (DSN, network meeting)*.

*“Good to transfer responsibility to the patient.” (DSN, network meeting)*.

### Giving and receiving support

Giving and receiving support represents the need for feedback on lifestyle changes. Feedback must be given at the right time, consider the patient’s specific needs, and avoid increasing the DSN’s workload. This generic category was divided into two subcategories: “Feedback” and “Timing”.

#### Giving and receiving support – feedback

Patients thought that the DSN was a key figure in helping them deal with diabetes. First, the DSN provided knowledge and feedback; and second, they felt they needed a personal relationship with their healthcare provider to help them focus on maintaining a healthy lifestyle.

*“This is of utmost importance. We need to meet the first time. We need the human touch.” (patient, focus group 1)*.

Moreover, patients wished to share their experiences with other individuals with T2DM. Giving and receiving support from others in a comparable situation was considered valuable, for example, when choosing suitable foods. That type of peer support could increase the potential of getting instant feedback when questions arise.

*“To meet in a group like this and talk and hear other people’s experiences. That’s not wrong either. (Patient, focus group 2)*

The DSN could see that their patients were a heterogeneous group, with varying needs for feedback. In general, DSNs used feedback to assign and review treatment goals. Furthermore, another important aspect of feedback was to provide a safety net, which could ensure that the patients knew how to act when serious symptoms arose. The DSNs used conversational skills to improve their understanding of the kind of support the patient needed.

*“I usually speak about the importance of treatment goals, both now, but also in five to ten years. This is one of our most important tasks.” (DSN, focus group 3)*.

Several suggestions were proposed for creating a DHI that could meet the need for feedback. For example, chatrooms could be set up between the DSNs and patients; digital group meetings could be organized; and a posting board that listed tasks for the patient to perform and individualized feedback from the DSN. Patients suggested that a DHI adapted to an individual patient’s specific needs would be more likely to succeed than a DHI that provided the same information for everyone. Thus, both the patients and the DSNs clearly expressed a need for an interactive system that could be used in collaboration with the usual care, rather than a standalone system that could not be accessed by their regular healthcare provider. Patients wanted a system that allowed feedback from their DSN, and DSNs wanted access to facilitate support for their patients.

*“It is a good thing to give homework. “Read this until next time”” (DSN, network meeting)*.

*“The patient is in control and the conversation is based on what they need. Then they can change at their own pace and it will be according to their life.” (DSN, focus group 3)*.

#### Giving and receiving support – timing

The patients thought that the timing of receiving information and support was important after a T2DM diagnosis. The emotions triggered often impaired the ability to process information about their disease. Thus, patients emphasized that it was particularly important for them to have an opportunity to process emotions related to the diagnosis, before starting the DSMES.

*“The brain can’t process information during the first medical visit. All advice needs to be given after a while.” (patient, focus group 2)*.

DSNs often felt that they lacked time during the first visit with a patient. Spending a lot of time repeating basic information limited the time spent on supporting and stimulating actual behavioral changes. It takes time to get to know the patient and provide individualized information. In an optimal setting, the DSN would meet the patient regularly over several weeks to establish contact and increase the patient’s understanding of T2DM. However, DSNs felt that this approach was not possible in their work setting, due to heavy workloads. DSNs also feared that a DHI application might require the prioritization of DHI operations over their other duties, which might further increase their workload.

*“There is always a lot of information [about food] at the beginning. I want follow-up calls but I don’t have time. Always fully booked.” (DSN, focus group 3)*.

*“Questions and answers must be handled during the allotted time and not continuously during the day, then it becomes a stressful moment.” (DSN, network meeting)*.

In a DHI, appropriate timing could be met by establishing asynchronous chatrooms, where patients could ask questions between medical visits to get advice about diabetes, but the DSN could choose to respond at times when their job assignments allowed. Additionally, DSNs wanted the patient to prepare in advance before attending medical visits to save time; this would allow the DSN to work on prioritized health areas. A DHI that was always available to the patient would allow patients to choose when to take part in diabetes education and when to start lifestyle changes.

*“I would like the patients to have read all the basic information about T2DM after being diagnosed at the doctor’s visit. Then, they can have relevant questions for me, and I could tailor the visit to save time.” (DSN, focus group 3)*.

## Discussion

This study aimed to explore patient needs after being diagnosed with T2DM, the needs of DSNs, treating these patients, and to elucidate how these needs identified by patients and DSNs might be met by a DHI for DSMES. We identified the overall theme: “overcoming the struggle of living with T2DM”, and this theme comprised two generic categories: (1) “learning and being prepared”, which comprised the subcategories “understanding” and “control and responsibility”; and (2) “giving and receiving support”, which comprised the subcategories “feedback” and “timing”. Respondents felt that these needs could be met with a DHI that was integrated into the patient’s regular care and was used in collaboration with the DSN.

The overall theme, “overcoming the struggle of living with type 2 diabetes”, could be expressed as ‘diabetes distress’, which is a frequent problem in T2D [20]. Diabetes distress is caused by thoughts of disease-related physical limitations, high self-management demands, unresponsive providers, and unsupportive interpersonal relationships, which are common problems when living with a chronic disease like T2DM. Diabetes distress can cause feelings of inadequacy, shame, and guilt, and it has been associated with inadequate self-management, which leads to, e.g., poor glycemic control [21, 22].

Notably, our results suggested that, from the patient perspective, diabetes distress was not sufficiently addressed by the DSNs. This deficiency was also suggested in previous studies [23]. A DHI could lead to stable, meaningful routines, and thereby, support the patient with T2DM. Alternatively, it may pose a burden to self-management, due to inherent technical challenges [17]. Nevertheless, a DHI could increase the emotional support needed by patients with T2DM, if it were integrated into regular patient care, highlighted the emotional aspects of living with a chronic disease, and facilitated collaboration between the patient and the DSN. These needs were also reported to be important in previous studies [15].

The patients in the focus groups desired more knowledge about diabetes. Information about lifestyle choices and healthy foods was deemed central in guiding the patient to making the correct choices, in specific situations. Furthermore, the DSNs wanted to provide clear, correct information to help patients become experts on their disease; thus, the information could focus on diabetes management, instead of repeating the basic facts. Importantly, more knowledge might not be sufficient to improve glycemic control [24]. Therefore, traditional didactive patient education, where the provider teaches patients about their disease, should be replaced by empowerment-based programs that support self-management. To achieve successful self-management, providers should promote the patient’s capacity to define the problems they are facing, make informed decisions about their diabetes management, set realistic goals, and define strategies to meet those goals. Despite this awareness, deductive patient education remains overrepresented [25–27]. To meet the needs of knowledge and understanding to support self-management, we propose that a DHI must be flexible, with adaptable feedback, based on the patient’s special needs. These criteria are supported by previous research [28].

We found that patients and DSNs had divergent views on responsibility. Patients felt that outside control would facilitate better compliance and the maintenance of behavioral changes over time. In contrast, the DSNs struggled with patients not taking on the responsibility of self-management. However, long-term behavioral change is challenging to most people. Nevertheless, self-determination theory claims that healthy habits, such as physical activity or healthy eating, are not maintained over time, when they are implemented in response to external pressure. Indeed, outside pressure undermines the development of an individual’s self-motivational resources, which are needed for successful self-management [29]. Therefore, healthcare providers should focus on helping the patient find internal motivation to regulate new behaviors more autonomously [30]. The use of a person-centered counseling approach could help the patient in this process [31].

The aspect of patient responsibility was further highlighted by the patients’ suggestion that they should collect more patient-generated data. That data could also be monitored by the DSN to facilitate self-management. They stressed that self-monitoring blood glucose levels could increase the sense of being in control of their disease, which could motivate the maintenance of healthy behaviors. However, this suggestion was contradicted by results from the ESMON study, which showed that self-monitoring blood glucose levels was not associated with any improvement in glycemic control, and instead, it reduced wellbeing [32]. Furthermore, the DSNs referred to current diabetes guidelines in Sweden, which generally do not recommend self-monitoring blood glucose levels [33]. We propose that a patient request for self-monitoring blood glucose levels might be best met with a patient-centered approach tailored to the needs of the specific patient.

A DHI could address the need for responsibility and control in diverse ways. For patients, it could display recurring tasks that guide the patient to finding internal motivation in making behavioral changes that support successful self-management over time. Furthermore, a means to report patient-generated data could increase the patient’s sense of control and deepen the DSN’s understanding of the patient’s disease, which could facilitate a person-centered approach. However, it is important that the DHI does not shift the responsibility of self-management from the patient to the DSN [34].

The patients emphasized that, to cope with feelings of insecurity, they needed feedback on self-care management from their DSN. This need was supported by results from other studies, which suggested that relational continuity is important for providing individualized support in self-management [35, 36]. Both patients and DSNs felt that the cornerstones in self-management were goal setting, practical recommendations on how to reach the goals, and regular revisions of the goals. Furthermore, patients discussed the need for peer support from other patients with T2D. Indeed, peer support may facilitate self-management and improve quality of life with T2D. However, peer support may not be very effective for improving blood glucose levels [37].

The need for support and feedback were linked to the concept of timing. From the patient perspective, self-management would be best facilitated by receiving instant feedback when a question arises. However, providing instant feedback is challenging for DSNs, who commonly struggle with time constraints that force them to prioritize different patient needs in everyday work. Thus, it is essential that a DHI is compatible with the resources available in the healthcare system [34, 38]. From the DSN perspective, the demand could be compatible with resources, if they used standardized feedback that only required minor adjustments for individualization, and if it could be delivered asynchronously or only at specific time points.

A strength of this study was that we included both patients and DSNs. It is important for digitalization to meet the demands of both patients and healthcare providers to improve health care. A DHI would not be used, if it was relevant to patients, but not implementable in regular care, or if it could be implemented, but did not meet patient demands [14]. Furthermore, the study aimed to elucidate two questions: what were the specific needs and how could these needs be met with a DHI? It was clearly more easy for the participants to answer the first focus group question than the second – “How these needs might be met by a DHI?”. A possible explanation might be that the patient participants had experienced deficiencies in their regular diabetes care but not the use of a DHI which therefore was harder to elucidate upon. Nevertheless, both questions provided relevant information from a clinical perspective. The purposive sampling provided a group of patients that included both sexes and different ages. In contrast, the majority of DSNs were female; however, this sex distribution was representative of DSNs in Swedish primary care.

This study also had some limitations. In a focus group setting participants might not voice their opinion voluntarily. It might be easy to agree with the majority and this is difficult to control for. On the other hand using focus group can illuminate different perspectives and participants enriches each other. To increase the transferability of the results, we broadened the group of DSNs by including data from a networking meeting. All patients in this study used computers and/or smartphones on a daily basis; thus, our findings may not be generalizable to patient groups with little experience with digital devices. To increase confirmability, the researchers were involved at different stages during the analysis process and in the interpretation of the results. Importantly, saturation was reached, because the same statements were recorded in both focus groups [39]. The DSN focus group gave depth to the discussion, and the DSNs from the networking meeting provided a broader picture; in addition, the latter DSN group confirmed the statements made in the DSN focus group.

Three members of the research team are working as resident physicians which might affect the participating DSN and the patients. To reduce the risk of bias researchers avoid participating in focus groups if treating a patient or having a professional relationship with a DSN.

One participant had had T2DM > 3 years and was incorrectly included in the study. This was discovered after this patient attended the focus group. We judged that this did not impact the results in general since it was only one participant.

We did not specifically address whether the research team’s personal beliefs and biases might have influenced the data collection process and analyses. Three of the researchers had positions as resident physicians in the primary health care, and therefore the experiences and beliefs of these researchers may have impacted the interpretation of the qualitative data. This has to be taken into account when reading the results and discussion of the findings in this paper. To reduce the risk of bias in the focus groups, predefined probing questions were included in the interview guide and a second researcher was present as an observer during the focus groups. Furthermore, doctor-patient relationships were avoided when the focus groups were arranged.

## Conclusion

This study showed that patients with T2DM and DSNs felt that a DHI for DSMES could meet the needs of both patients and providers. A DHI might enable the patient to be more active in understanding T2DM and shift the responsibility of setting and striving towards treatment goals from the DSN to the patient. Furthermore, a DHI might increase the ability of the DSN to provide person-centered care and more support to the patient. However, the DHI must be integrated into routine care, and the demands of using the DHI should be balanced with the resources available in the healthcare system.

## Data Availability

The transcribed interviews from focus groups are maintained by the researchers and may be available upon request, when in accordance with Swedish law. If data from the study is requested, please contact Frida Jarl.

## List of Abbreviations

DHI: digital health intervention
DSMES: diabetes self-management education and support
NDR: National Diabetes Registry
PHCC: primary healthcare center
DSN: Diabetes specialist nurse
T2DM: type 2 diabetes
